# From waste product to blood, brains and narratives: developing a pluralist sociology of contributions to health research

**DOI:** 10.1111/1467-9566.12715

**Published:** 2018-03-01

**Authors:** Anne‐Marie R. Boylan, Louise Locock, Laura Machin

**Affiliations:** ^1^ Health Experiences Research Group (HERG), Nuffield Department of Primary Care, Health Sciences University of Oxford UK; ^2^ Health Services Research Unit University of Aberdeen UK; ^3^ Lancaster Medical School Lancaster University UK

**Keywords:** blood donation, biobanks, narratives, organ donation, secondary analysis

## Abstract

The aim of this paper is to examine the meaning of the concept of donation in health research. Drawing on a set of narrative interviews with people invited to donate biosamples for research and a range of other studies, we identify several conceptual themes that speak to the complexity of the current landscape of critical thinking about donation. These conceptual themes are: the language of ‘donation’; a hierarchy of biosamples; alternative informational value; narratives as donation; coincidental donation, convenience and degree of invasiveness; and rights, consent and benefits of research participation. We call for a reconceptualisation of research donation to encompass not only the numerous types of sample readily classed as donations, but also other types of data and contributions, including narrative interviews, psychometric data, patient‐reported outcome measures, record‐linkage, and time and effort. We argue for the development of a pluralist sociology of research donations, and suggest that a ‘sociology of research contributions’ might better capture this complexity.

## Health research ‘donations’

In this paper we examine the meaning of ‘donation’ in the health research context, looking not just at biosamples but other forms of personal donation to research. In biomedicine research donation is primarily understood as biosamples, bodily fluids and tissue such as urine, blood, brain, tumour, and embryos. Regulatory frameworks and academic literature have tended to treat all types of sample as equally significant, even though this may not adequately reflect the real‐life views and motivations of research donors.

Alongside biosamples, people often give consent for personal biometric and lifestyle information to be collected and used, and for medical record linkage. However, such information is rarely considered through a ‘donation’ lens; rather the debate is framed in terms of data protection and anonymity.

People contribute to interdisciplinary health research in a range of other ways, including, for example, committing time and effort, agreeing to experimental treatments (such as in clinical trials), taking part in surveys, completing patient‐reported outcome measures and giving interviews.

We suggest that a sociology of ‘donation’ as a single entity is problematic. We seek to reconceptualise research donation, extending the current ‘hierarchy of donation’ identified by Machin and Cherkassky (2015) to encompass these various forms of contribution.

### Biosamples and donation

Theorising donation for research takes place against a backdrop of two major competing influences. On the one hand, Titmuss's (1970) analysis of blood donation as a ‘gift relationship’ looms large. There is long‐standing debate as to how far Titmuss departed from or acknowledges the relevance of traditional anthropological understandings of the gift relationship as a network of reciprocal obligations (Rapport and Maggs 2002, Tutton 2002). Whatever Titmuss intended, his emphasis on the ‘free human gift’ with no immediate expectation of return has been co‐opted beyond therapeutic donation (i.e. donation of blood and organs for treatment purposes) into medical research policy, our focus in this paper.

Altruism has been promoted by medical research agencies as the underpinning principle for biosample donation (Kanellopoulou 2009, Machin and Cherkassky 2015). People are urged to donate partly to fulfil their desire to help others, but also because this will promote the kind of community where their donation would be reciprocated (Moorlock *et al*. 2014). Donation is less about individual gratification and instead about a community duty (Whitfield 2014) that donors can relate to, generating a sense of solidarity within unknown others in a ‘community’. For example embryo donors believe they are supporting infertility research, and by default, the benefactors of their donated embryos are not completely anonymous due to the imaginatively shared experience of infertility (Scully *et al*. 2012).

The very word ‘donation’ exemplifies this emphasis on altruism – implying a one‐way philanthropic act (see ‘the language of donation’ below). The language of gift has been actively promoted by government and health agencies to secure human tissue for various uses (Shaw 2008, Tutton 2004). On the other hand, concerns about exploitation of donated material have led to a body of socio‐ethical literature promoting personal property rights over biosamples, exemplified by the polemical titles of Andrews and Nelkin's (2001) *Body Bazaar: the market for human tissues in the biotechnology age* and Scheper‐Hughes's (2001) ‘Bodies for sale – whole or in parts’. More recently, Swiss participants expressed anger that they would not be compensated for giving up their embryos and that through donation researchers were getting something for nothing (Scully *et al*. 2012).

Following the Alder Hey scandal in the UK during the 1980s–1990s and the disputed research use of cancer cells derived without consent from the late Henrietta Lacks in the US (Hudson and Collins 2013), reasonable concerns about consent and future use of samples have evolved into a socio‐ethical presumption that all body parts deserve special respect and protection. However, empirical literature suggests the public may not see things through the same lens as theorists. In previous work, Locock and Boylan (2016) explored whether patients and healthy members of the public contributing to biobanks saw donation as a gift; while the term resonated for a few people, for others it seemed exaggerated and even slightly ridiculous. While people were concerned to some degree about the governance of research and future use of samples, there was little evidence of wanting to retain property rights or seeing samples as a special part of oneself. This is consistent with reviews of attitudes to donation (see Hoeyer 2008, Lipworth *et al*. 2011), suggesting neither a gift nor a property rights model is a good ‘fit’ with public views.

Importantly, however, Hoeyer (2008) argues that people's views vary depending on the type of tissue being donated and the position of the donors in relation to the research project (for example whether they are ill or healthy). He also suggests that donors can simultaneously hold apparently conflicting views that a blood sample is ‘*both* a few drops of blood of no consequence *and* important as “part of” the donors’. (Hoeyer 2004: 98, original italics). Bahadur *et al*. (2010) confirm that context and purpose of donation, the extent to which tissue is seen as valuable or waste product, and the identity of the potential recipient all play an influential role in donor attitudes towards both therapeutic and research donation, specifically donors’ decisions to donate or not.

Machin and Cherkassky (2015) claim that the context and purpose of donation results in a hierarchy forming based on the value of a body part or outcome acquired through, and attached to, the process of donation. As a result, ‘all donors and their donations are therefore not equal’. (Machin and Cherkassky 2015: 146) Here, we aim to explore and broaden this hierarchy. By stabilising the hierarchy so that one purpose of donation is the focus, that is, research, we are able to draw attention to the significance of time and effort within donation. Ontologically, time and effort have rarely been acknowledged as ‘donated’, perhaps as a product of what has previously mattered in health research, namely the physical body (discussed further below). The creation of a donation can require varying investment from a donor according to what is being donated; for example, compare the physical and emotional labour for embryo donors to the convenience of donating a urine sample. This element of generating a donation demonstrates the range of emotional attachment donors can have towards their donations, raising the question if donating a brain tumour is somehow lower in the hierarchy than donating aborted foetal tissue, based upon the initial (moral) origins of the donation. In essence, the epistemological status of the donation can vary according to the ease with which the donation is collected and acquired, for example, surgery, blood test, and the health status of the donor, collected as part of ‘care’ received or optional and chosen by a healthy donor. By focusing on the time and effort considerations to facilitate research donation, we argue that a broader view is required for ‘what’ can be ‘donated’, with implications for how we understand and make sense of ‘donation’ today.

### Beyond biosamples ‐ other forms of personal research donation?

We seek to widen the debate on research ‘donation’ away from its traditional dominant focus on body parts and fluids, to consider other ways that people may make deeply personal contributions to health research, including narratives, personal data, and time and effort. These differing forms of donation are rarely considered together in clinical research discourse, reflecting a biomedically driven conception of what matters in health research – namely the purely physical body – rather than a socially driven, experiential understanding of the self. Drawing on the work of Merleau‐Ponty, who argued the inextricable link between mind and body, we contest this current conception, propose that it is lacking and argue for a broader definition.

Since the seventeenth century, the body has come to be viewed through the scientific lens, as ‘a material object whose anatomical and functional properties can be characterised according to general scientific law’ (Leder 1990: 5). The contents and processes of the mind, and experiential information collected by the body over time have often been relegated in much medical research to a second class of subjectively‐informed knowledge, compared with objective, scientifically‐generated knowledge. Therefore, it is unsurprising that clinical research has primarily focused on the body as a resource for measurable, observable biosample donations.

Merleau‐Ponty (1962) rejected Cartesian dualism, the notion that the body is an object like any other, and argued that human bodies are distinct from other objects and provide a means for communication with the world. Thus, the human body is not only a set of donatable parts, masses, fluids and samples, but a complex organism interacting with its environment to produce an ongoing store of experiences. These experiences are gathered through physical communication with the world – seeing, hearing, sensing, tasting, touching and, through a series of neural processes, are given emotional colour, interpreted, converted to memories and stored for future recall. The body is the meeting point between the person and the world, and is our means for having a world; as such, all experience is embodied. So the body as ‘something one “has” … has to be augmented with an understanding of the body as something that one “is” … the medium by which we are able to live our lives, and establish and sustain an individual, reflexive (capable of self‐reflection) identity’ (Fox 2012: 49)

We therefore suggest that ‘donations’ of experiential information or personal data are in fact embodied as they require a state of embodiment to exist. When making physical donations to research, people also donate time and effort – that which is directly involved in the donation process and that which is involved in getting there (e.g. travel, preparation, fasting, childcare arrangements, annual leave). Personal information (e.g. cognitive function, lung capacity, height and weight) and access to medical records also pertain to the body. For example, when the body was born; its age; its previous history of interactions with medical interventions, procedures, drugs and vaccines. Future personal information, in the form of medical record linkage, may link bodily disease to biosamples many years down the track. Tutton (2002: 537) contrasts what he calls the ‘corporeal’ economy of blood transfusion, in which blood itself is the valuable life‐saving thing, with ‘the informational economy of research’ in which blood is only valuable as a route to getting information about DNA. Therefore, more attention on informational ‘donation’ is warranted.

But not only is our body the storage facility containing our repertoire of experiences, it is the means through which we verbalise our experiences. As Merleau‐Ponty (1964: 5) asserts; ‘The body is much more than an instrument or a means; it is our expression in the world, the visible form of our intentions’. When people take part in narrative research, they are contributing opinions, values, thoughts, reflections and emotions derived from their physical and mental experiences. We suggest that the hierarchy of research donations should be expanded to include donations of the mind as well as of the body.

## Methods

Our purpose in this paper is to stimulate further conceptual discussion of future directions for research within the sociology of donations field. While this article is intended to be primarily reflective, we feel it is important to ground any such reflection in the views of real people, rather than hypothetical ethical or theoretical considerations which may hold little meaning or relevance for research participants. In this we follow Shaw's (2015: 952–3) methodological approach, aiming to ‘expand the conceptual toolkit’ in a way that is ‘both analytical and empirically oriented, drawing on research that links a series of qualitative sociological studies’. Our analysis is primarily what Heaton (2004: 38) defines as ‘supra‐analysis’, secondary analysis which ‘transcends the focus of the primary study … examining new empirical, theoretical or methodological questions’. We draw in particular on a narrative interview study conducted jointly by authors one and two in 2010–11 with 21 people (Table 1) who had been invited to give biosamples for research (approved by Berkshire Research Ethics Committee Ref 09/H0505/66). The aim of this study was to explore people's experiences of, and attitudes towards biobanking and biosample donation, both for specific local research studies and for national repositories such as the UK Biobank.

**Table 1:** Sample characteristics, biobanking study

Participant	Type of biobanking/sample	Gender and age
01	Blood and tumour samples, cancer; blood, saliva and urine, population biobank	F, 55
02	Blood and tumour samples, cancer	M, 58
03	Healthy volunteer, population biobank (declined)	M, 43
04	Healthy volunteer, blood samples and fat biopsies, diabetes research (declined population biobank)	F, 49
05	Blood samples, cancer	F, 52
06	Healthy volunteer, blood, saliva and urine, population biobank	F, 49
07	Healthy volunteer, blood, saliva and urine, population biobank; blood samples and fat biopsies, diabetes research	F, 52
08	Blood and liver tissue samples, Hepatitis C Virus	M, 54
09	Blood and spinal fluid samples, Motor Neurone Disease; blood samples, MND DNA bank	F, 56
10	Blood samples, Hepatitis C Virus	M, 66
11	Healthy volunteer, blood samples and fat biopsies, diabetes research (accepted and declined on different occasions)	M, 49
12	Blood and spinal fluid samples, Motor Neurone Disease	M, 63
13	Blood samples and post‐mortem brain bank, Motor Neurone Disease (spinal fluid samples declined)	M, 61
14	Healthy volunteer, blood samples, stroke study as family member control; blood, saliva and urine, population biobank	F, 62
15	Blood and urine samples, high risk pregnancy; healthy volunteer, blood, saliva and urine, population biobank	F, 45
16	Blood samples, Motor Neurone Disease (spinal fluid samples declined)	M, 54
17	Blood and urine samples, high risk pregnancy	F, 37
18	Blood samples, Hepatitis C Virus	M, 49
19	Healthy volunteer, blood and umbilical cord blood samples, pregnancy biobank	F (age withheld)
20	Healthy volunteer, blood and urine, population biobank	M, 33
21	Healthy volunteer, blood and urine, population biobank	F, 44

However, we also draw more broadly on a linked series of previous studies of medical research participation and involvement, examining people's experiences of and motivations for such activity (Crocker *et al*. 2016, Locock *et al*. 2017, Locock and Smith 2011a, 2011b). As we extend our discussion to non‐bodily forms of research donation we draw also on our wider experience of, and reflections on conducting narrative health research in the UK, including primary and secondary analysis. Ethical approval for all these studies included consent for secondary analysis.

All three authors met for two days of discussion to workshop the data, reflect on the literature and develop the conceptual themes.

## Conceptual themes

From our reflections on the literature and the empirical data, we identify several conceptual themes which explore the various dimensions of research donation and extend our thinking beyond traditional models: the language of ‘donation’; a hierarchy of biosamples: from tumour to blood, brains and bodies, from waste to value; alternative informational value; narratives as donation; coincidental donation, convenience and degree of invasiveness; and rights, consent and benefits of research participation.

## The language of ‘donation’

Language both reflects and shapes how researchers and participants understand the purpose and act of donation, and the nature of what is donated. Examining the language used can thus shed light on underlying assumptions. The very word ‘donation’ is problematic, conjuring up an image of charity, voluntarism and selfless giving to an imagined or ‘fictive’ other (Whitfield 2013). ‘Donor’ similarly calls to mind both philanthropy and – in the medical context – the therapeutic act of the blood donor and organ donor. Whitfield identifies the use of ‘donor’ as a wartime propaganda device in response to the marketing challenge of attracting people to give blood to newly collectivised, mechanised and impersonal blood banks.

Drawing on the analogy of donating unwanted items to a charity shop, O'Neill (2009) suggests that three criteria must be met for the word ‘donation’ to apply:

1. the item is surplus to our needs;
2. we can easily replace it if needed, if the surplus does not last; and
3. the recipient is an anonymous needy individual.

She argues that although therapeutic blood donation does seem to meet these criteria, generally ‘bodily donations are obviously different from giving to Oxfam’ (O'Neill 2009: 154). In fact in our studies, Jane^1^ explicitly compared giving tumour samples for research to recycling unwanted Christmas gifts ‘through the charity shop’. O'Neill focuses primarily on therapeutic rather than research donation but does consider dead body donation for anatomical research. This she concludes does not meet the criteria, because although the body may be surplus to the individual's needs, families may still feel a ‘need’ for an intact body and the idea of it being easily replaceable makes no sense. Furthermore (echoing Whitfield) she suggests the donation to an intermediate organisation, such as a university, removes even the ‘fictive’ needy individual from the equation.

In a previous paper (Locock and Boylan 2016), we noted that while the verbs ‘give’ and ‘donate’ were used readily in people's talk about biosamples, the noun ‘gift’ seemed to hold more substantial meaning as something deliberate and almost ceremonial, distinct from the everyday word ‘giving’. In our studies Debbie laughed at the idea that giving a urine sample for research might be a gift. She mimed the act of handing over a present, saying sarcastically, ‘Hello, happy birthday’. While this might not be true of all types of sample, and people frequently distinguished blood from other types of sample (see ‘a hierarchy of donations’ below), it underlines the complexity of seeing ‘donation’ as a single process with a common meaning for all parties. Mahon‐Daly (2012: 234–5) in her study of blood donation notes different attitudes in people using ‘donate’ or give’, arguing that language of ‘donating their blood was linked to more expression of altruism … Donating infers a bestowing action, with the freedom for the recipient to do what they want with the gift. Giving may be more like “letting someone have”’. However, her suggestion that the latter ‘implies that the giver wants to have a say in the gift after it has been given’ does not resonate with our findings, which suggest ‘giving’ was a much more casual act.

In the research donation context, we reflect that nouns such as ‘sample’, ‘biosample’, ‘material’ and ‘tissue’ may also generate particular meanings, redolent of objectifying scientific practice. They may exercise a depersonalising effect, distancing the item to be scrutinised from the person. We are unable to think of any parallel collective word in therapeutic donation, where the discourse is more likely to be around what specifically is given (blood donation, organ donation, bone marrow donation). The verb ‘harvest’ occurs both in official information and in the talk of donors. While the implication of fruitful productivity has a certain positive note, ‘harvest’ also conveys an impersonal and almost industrial process.

Narratives and personal medical data are rarely conceptualised using the same language as that used to describe biosamples, which may minimise their potential for being considered as donations. But people routinely talk about ‘giving’ an interview, and interviews, personal information and information contained in medical records, conceivably meet O'Neill's (2009) criteria for donations. We explore this further in ‘alternative information value’.

## A hierarchy of biosamples: from tumour to blood, brains and bodies, from waste to value

In line with Hoeyer's (2004) and Bahadur *et al*.’s (2010) conclusions that the type of sample affects the attitude of the donor, we have also found a spectrum of concern, from distasteful waste product at one end to valued, life‐giving material at the other. Perhaps the most extreme form of waste product is tumour, which is not just passively unwanted in the sense of being of no value, but actively unwanted in the sense of being a harmful ‘thing’, invading the body.

In cancer and other serious diseases, donation to research may be experienced as the taking back of control over one's body and illness. The excised tumour – which would otherwise simply be thrown away with the rubbish – is harnessed for good, perhaps diminishing its threatening and repellent nature. It is certainly ‘surplus to requirements’, as O'Neill (2009) suggests, though the ideas that anyone would want to replace a tumour or that an ‘anonymous needy individual’, in this case a scientific researcher, is waiting for it are less relevant. Nonetheless, particularly in the context of possibly hereditary cancers, participants could anticipate a direct familial benefit for their own descendants, as well as a more diffuse benefit to ‘other people like me in future’.

Urine and saliva may fall further along the continuum, unvalued and faintly distasteful, but not as toxic as tumour samples. Urine, in particular, is difficult for people to conceptualise as valuable to anyone other than a researcher. People may struggle to see why donating something that would otherwise be flushed down the toilet could ever be considered controversial, risky or indeed special. Saliva can be understood as a source of valuable DNA information (for example in forensic dramas or paternity cases); even so in our participants it evoked remarkably little concern.

At the opposite end of the spectrum of live biosample donations lies blood, with all its physical and symbolic significance. Blood keeps us alive; it represents familial bonds (‘blood relatives’); and in the Judaeo‐Christian tradition it holds sacramental and sacrificial meaning. Blood‐related metaphors permeate the language: we talk of something ‘coursing through our veins’ or being ‘in our blood’, conveying something essential to our identity. Yet even in the case of blood, people may take a very pragmatic and unconcerned attitude to its use for research. Gerry, a participant who had Motor Neurone Disease (MND), said he wouldn't think twice about ‘a few phials of blood’. Jim, who had hepatitis C, said he saw blood in a similar way to other tissue samples he had given over the years, drawing on an objectifying metaphor of the body as something he viewed ‘quite mechanically’ and highlighting the ability of blood and cells to replace themselves.

However, our participants’ accounts suggest that keeping a clear separation between research and therapeutic donation of blood can be hard. Blood donation – perhaps because of Titmuss or many media campaigns – seems strongly associated in the public imagination with therapeutic donation and saving lives, and narratives often slipped into this territory. Debbie, for example, having laughed at the idea of urine as a gift, drew a specific contrast between blood and other bodily fluids, drawing on her own past history of both giving and receiving blood for therapeutic purposes.

Asked specifically whether this applied to research blood donation, she confirmed that she still saw it in these terms ‘because I'm always amazed at how much information they get out of how little’. We return to this in ‘Alternative informational value’.

Research biodonations after death – either specific body parts which can only be donated after death, such as brains and corneas, or whole bodies for scientific research – represent a very different form of donation to ‘live’ biosample donation. Organs such as hearts, eyes and brains carry an emotional investment which may make them “troubling to donate compared with ‘lesser’ body parts or materials even when the donation takes place post‐mortem.” (Bahadur *et al*. 2010: 871).

Duncan had already signed up to give his brain to an MND research ‘brain bank’. He said it was an obvious decision – ‘a bit of a no‐brainer’. Steve, a cancer patient, could not understand why ‘people get all precious about it’. Like Duncan, he took a pragmatic view of death and waste, commenting that the body is ‘either burnt or it rots, one of the two’, so it might as well be ‘put to good use’.

Others expressed uncertainty and in some cases a degree of squeamishness. Bruce found brain donation and his own death ‘an odd thing to think about’. Here and in other cases the squeamishness seemed less about the specific body part and more the sheer fact of mortality and the dead body. Aileen held a similar view that ‘when you're dead, you're dead’, but identified a certain ‘horror factor’, perhaps akin to Midgley's (2000) ‘yuk factor’ in relation to biotechnology. Debbie, although willing in principle to donate her brain, found the idea of a ‘brain bank’ ‘really horrendous’, and joked ‘I would like to withdraw a brain, please’.

Alongside the brain, donations involving the eyes and corneas evoke similarly mixed emotions. Anne, a healthy volunteer, contrasted her own pragmatic view about using body parts after her death with that of family members, who were ‘very happy to donate some things but not other things’, explaining ‘lots of people can't bear the idea of donating their eyes’.

Whole‐body donation after death is also a source of conflicting and ambivalent views. Jane, who had not thought about it before, felt brain donation for research would be acceptable, but felt less sure about whole‐body donation, especially use of her body by medical students and the ‘feeling of being scrutinised’.

Ellen, by contrast, was actively considering donating her whole body to medical science. She cried as she recalled a TV programme about how one medical school holds a ‘service of thanks every year for the families of those who have donated their bodies … it makes me even more inclined to do it’.

In the few years before the fieldwork was conducted, Gunther von Hagens’ ‘Bodyworlds’ exhibition of plastinated bodies had visited the UK and a human dissection was shown on TV. This seemed a step too far for some people. In Steve's words:

I wouldn't like the indignity of being dissected in public like that German guy did recently … An organ, specific organs I wouldn't have a problem with.

A common thread in these views is the importance of bodily dignity and a sense that the body is the self, requiring respect and shielding from inappropriate scrutiny. As Steve implies, this is in contrast to ‘specific organs’ – as in living donation, people express nuances and gradations within donation after death, which are rarely reflected in the media or the academic literature.

## Alternative informational value

Earlier we noted how Debbie articulated that the value of blood samples for research lay not so much in its life‐giving properties but in the information derived from it. This prompted her to somewhat re‐evaluate her views about the informational value of urine. This resonates with Machin and Cherkassky's (2015) emphasis on how the purpose of donation may generate different meanings. Yet intuitively Debbie could not believe urine was likely to yield as much valuable information as blood. Informational value is not necessarily immediately obvious.

While biosamples are traditionally seen as ‘donations’, other forms of information are differently conceptualised and differently valued, not even featuring in the assumed hierarchy of donations. When donating to the biobank, people are usually asked to consent to linking their data to their medical records and to provide accompanying personal information. Linking to medical records results in a holistic picture of current health to be established, allows for information to be tracked over the life course and for the development of future information through research, for example, genetic marker studies. Other information might include questionnaires about lifestyle, psychometric measures of cognitive functioning, and other biometrics, such as measurements of weight, height, BMI, blood pressure, etc. The participants recognised the value in this information: Janet explained that although it is not hard and fast like that gleaned from a blood test, it is important to give honest answers.

It's very tempting to exaggerate where you know it's good to do it and underplay it when it's good not to, like the alcohol. But … unless we give honest answers here it's not going to help the research. And when it comes to things like your blood test, then you can't fake that, so why fake the other parts of it?

Biosamples, seen as objective, scientific samples containing encoded information that requires careful work to extract, are judged inherently more valuable than self‐reported information, which is considered likely to be inaccurate. However, the linkage and triangulation of a range of data is more valuable than any single sample alone, consistent with Hoeyer's assertion that more value lies in the information derived from the sample than in the sample itself. This reinforces the case for widening the definition of donation beyond biosamples alone.

## Narratives as donation

Also neglected from the traditional hierarchy of donation, the value held by narrative interviews donated to qualitative health research studies is distinct. Biosamples and supplementary information require each other to extract maximum value, whereas the value of a narrative is more self‐contained and intrinsic, and both need interpretation and analysis to achieve their full value. Mazanderani *et al*. (2013) extend Mitchell and Waldby's (2010) notion of biovalue in relation to tissue samples to the value which may be derived from narrative interviews. This ‘biographical value’ may include value to social scientists, clinical researchers and doctors, in understanding better the embodied experience of illness, but also value to the self and to other patients. Narratives (particularly those of celebrities’) could also raise the profile of a disease and generate pressure for research funding and better treatment.

In donating an interview to health research, people may consent to the archiving of their interview for use in secondary analysis, meaning that after their participation has ceased, potentially after their death, their narrative still exists, and still has a purpose for social science. Just as donated bodies can be used in a variety of settings (education and training, research, organ harvesting, etc.), interviews can be repurposed to answer different research questions. Equally, donated interviews should be treated with the same dignity with which people expect their bodies to be treated after death, starting with informed consent and ensuring they are fully informed about the potential future uses of their donated experiences.

Stories of personal experience may contribute to research in ways which go beyond data collection and research participation. In the UK, the involvement of patients and members of the public (‘PPI’) in research has become an accepted part of research process. This may include reviewing information sheets to setting research priorities and designing research studies. Patients bring their physical and emotional experience of illness and treatment to bear on research to improve its relevance and acceptability for other people. Although there is a substantial literature on motivations for PPI, which includes discussion of altruism, voluntarism and the potential to help others, this has not to our knowledge previously been couched in terms of ‘research donation’.

Returning to O'Neill's criteria for how we understand donation, first, interviews and other additional information are surplus to our needs. Second, such donations do not need to be replenished – stories of personal experience can be retold or reinterpreted and giving personal information does not create a deficit. Finally, many people who give their stories say they were motivated by altruism or to ensure others have improved experiences (Lucius‐Hoene *et al*. 2013, Peel *et al*. 2006). Michael, a biobanking participant with Hepatitis C, was happy when the consultant asked him to tell his story to a group of medical students:

I poured my heart out to them. One in twelve people have this horrible, evil little virus, and I want to do as much as I can to help others and to help this study.

## Coincidental donation, convenience and degree of invasiveness

As the above suggests, another factor affecting people's attitudes to donating biosamples is not just the nature of the sample being taken but also the level of burden, discomfort, time and effort involved, and indeed perceived personal benefit, factors which inevitably affect contributions to other types of research, such as narrative interviews or indeed clinical trial participation.

People in our studies providing research biosamples during the course of their treatment commonly regarded this as uncontroversial and were unconcerned about future uses. One said, ‘it's not as if you've got to go into hospital for special surgery or to have extra blood taken’. Even if extra visits are required, this may not be seen as particularly inconvenient. Ellen, who gave additional blood and urine samples during her IVF pregnancy, recalled the research midwives being ‘terribly apologetic’ that this would involve an extra blood and urine test every month but she was ‘very, very happy to do all of this’.

We also spoke to several people who had been invited to take part in the UK Biobank, involving a two‐hour visit to a special UK Biobank centre. This degree of inconvenience was certainly off‐putting for some. Aileen (a frequent participant in diabetes research) explained that a letter came with a fixed appointment time and it ‘just didn't fit in with what was going on at that time’ so she opted out.

Yet her commitment to the diabetes research centre was extensive and often involved lengthy visits which she said she enjoyed. Others took part willingly in the UK Biobank, although – as is often the case with research participation – not necessarily having read the leaflet fully.

At the more invasive end of the spectrum, four of our participants who had MND had contributed to an MND biobank which included giving spinal fluid samples via lumbar puncture. Such an invasive procedure creates a wholly different experience of donation. In this case participants were offered the option to take part in other aspects of the study, but omit the lumbar puncture, which Duncan and Gerry did. Duncan previously had a bad experience of it, and Gerry explained that although he did not normally mind needles and blood samples, ‘It was just that one area of the body I just didn't want them to touch’.

By contrast Sam agreed to have a lumbar puncture, saying ‘I have a good pain threshold and my one thought always is, “I'm going to help, no matter how uncomfortable, how long it takes”‘.

We also spoke to several healthy participants in diabetes research, some of whom consented to invasive procedures (being injected with adrenaline and then having fat biopsies taken from the abdomen and thigh). Aileen had withdrawn from one study which required a blood sample from an artery in her leg because it was too painful:

It did put me off slightly at the time … Because they've got more invasive as they've gone on you do start to think, “Do I really want to go through this?” But having said that, if the letter came through next week or in a month's time, I'd probably do it again.

Motivation to donate biosamples in our studies was often tied to people's jobs (e.g. Aileen was a healthcare assistant) or family history. Another regular participant at the diabetes research centre had a family history of heart disease and high cholesterol, and found the regular blood tests reassuring. As the son of a pathologist, he was familiar with biosample research. Commonly people used terms like ‘giving back’ to the NHS for care received, by themselves or family.

Willingness to get involved in such invasive testing may thus depend on a range of factors, including perceived personal health and other benefits; familiarity with healthcare and health research; a sense of moral commitment and diffuse reciprocity; perceived degree of risk or discomfort; and severity of illness. Arguably any research donation requires a certain amount of time and inconvenience, and the impact of this should not be minimised. Time getting to the appointment may be invisible to researchers. One UK Biobank participant said she put a fruitcake in the oven before heading to the appointment, in the belief (having not read the leaflet) that ‘I was going to pop along, give a blood test, answer a few questions and whip away. And if it said two hours would I still have done it?’

Time and effort must be brought into the discussion about donation, and not solely when thinking about barriers and facilitators. Widening the definition to include these factors encourages researchers to be mindful of what donation entails and ensures that the donor is respected and credited for every aspect of their participation. In comparison with lumbar punctures and other physical donations, narrative donations may perhaps seem minimally invasive on the surface. However, participating in an interview requires time, the amount of which is often indeterminable at the outset, and mental effort – recalling and describing past experiences, worries, thoughts, and managing the emotional labour involved in the process. Despite being seen as ‘inherently innocuous’ (Peel *et al*. 2006), narrative interviews can focus on sensitive topics that invoke a range of emotions; they can be enjoyable, but can also arouse feelings of anger and distress (Lucius‐Hoene *et al*. 2013, Peel *et al*. 2006,). The specifics of the time and effort involved in narrative donation, requires us to think differently about according them a position in the hierarchy of donations.

## Rights, consent and benefits of research participation

A consistent theme across our studies (including participation in clinical trials and cohort studies as well as biobanking) has been not just willingness to participate in research, but active enthusiasm for doing so and a strong sense of personal gain. This is not simply a ‘therapeutic misconception’, as it is often described, but rather a rational assessment of a wide range of personal benefits, including enjoyment and satisfaction, learning about science, enhanced relationships with a clinical team providing your care, and increased monitoring or feedback about one's health. (Locock and Boylan 2016, Locock and Smith 2011b). Over several studies, a theme of disappointment has emerged that the right to be told about research opportunities in the NHS Constitution is not always enacted.

This assessment is underpinned by trust in regulation and in the source of the invitation to take part. This is not to say that people feel information and consent are unimportant; rather many feel they should have a choice about how much depth they want to go into, and that if they wish to make their decision based on cursory reading of the information that is their adult right. In saying this, they mirror the behaviour of most of us when faced with lengthy terms and conditions – they tick the box and move on (Bakos *et al*. 2014). Of course some people want to read every detail, and some people are inherently cautious about sharing their data. The phrase ‘Big Brother’ was used more than once in connection with the UK Biobank and suspicion about the use to which samples might be put in future. But many people seem relaxed about this, particularly in the case of ‘waste’ material such as tumour or urine. We have found little evident sense of property rights, and considerable concern about burdensome over‐reactions to situations such as Alder Hey, as Bruce argued:

I never understood all this fuss that came out – I know what they did at Alder Hey wasn't transparent, but I don't think it was wrong …This incredible bureaucracy that was loaded on the NHS to track down body parts …You get the impression that any bit of your body they take, you have to sign a consent form or you can expect to ask for it back next year … I'm not hugely attached to my body and I hope nobody else will be after I'm dead, and certainly to the extent of lumbering a stretched NHS with extra costs just for a bit of … I mean, I trim my toenails and they go in the bin. [laughs]

Taking part in narrative interview studies in health research certainly offers potential for personal benefit. In their study about participation in qualitative interviews, Lucius‐Hoene *et al*. (2013) reported that participants found the experience exceptional as they had an opportunity to talk uninterrupted about their condition and to be taken seriously by the interviewer. The interview presented an opportunity to reflect on their feeling about their illness and learn about themselves. Many of their participants found it an important and deeply moving experience, describing it as a liberating and pleasurable event that made them feel valued and renewed their sense of engagement with their lives. But for others it was a more negative experience, causing them to feel anger and distress. For a third group it did not seem to hold any positive or negative significance. This lack of significance chimes with the notion of interview participation as ‘inherently innocuous’ (Peel *et al*. 2006).

These experiences of interview studies resonate with how people talk about their experiences of donating biosamples: donation can be meaningful and beneficial; or donation may not be particularly significant and may be easy and uncontroversial to do; and it can occasionally be negative, perhaps experienced as burdensome, invasive or painful.

## Concluding remarks

As Bahadur *et al*. (2010: 872) argue, ‘one of the significant limitations of principalist, bioethics‐based regulatory governance is that … it necessarily presupposes that all donors have a standard set of concerns and moral values’.

In this paper we have added to the evidence that such a ‘one‐size‐fits‐all’ understanding of research donation is not helpful. We argue for a more nuanced understanding of biosamples, but also crucially that other forms of research contribution, including narratives, lifestyle and biometric data, agreement to take part in a study and involvement in research design, can all be conceived of as forms of embodied donation.

Biosample donation takes widely differing forms with very different consequences for individual donors; yet much of the ethical literature and research ethics processes treat all bodily samples as equivalent – equally risky, equally concerning, and equally in need of stringent conditions. Equally, relatively low‐risk research studies using qualitative methods or gathering self‐report information are treated to similar levels of ethical scrutiny. This discourse takes little account of the real‐life responses of actual participants. Hoeyer (2010) has challenged attempts by scholars, faced with contradictory data about people's attitudes, to find an underlying consensus, and establish a single universal principle for conceptualising the relationship with donors. He queries whether generic terms such as ‘biobank’ and ‘donor’ are fit for purpose in capturing this variety of motivations and wishes.

It is easy to argue that casual attitudes to donation among patients result from simple lack of awareness; certainly in interviewing or gathering self‐report information it is possible to create more anxiety by asking questions, and presenting scenarios where the potential for harm is foregrounded. The very act of questioning people or involving them in a hypothetical deliberative process may plant a seed of doubt that their judgements have been under‐informed and they ‘ought’ to think differently as responsible citizens (Hoeyer 2003). But a counter‐argument – that people are mature decision‐makers who have a right to take a casual attitude if they wish – is rarely made. The right to donate, the right to trust in researchers and research regulation, and the right not to have to read lengthy, impenetrable and risk‐averse participant information get little attention compared to the right to anonymity, and concerns about data protection, bodily integrity and autonomy.

Hoeyer (2010) notes a move to adopt the term ‘participant’ rather than ‘donor’:

to acknowledge the ongoing mutual nature of the relationship … and to avoid connotations to an over‐and‐done‐with ‘gift’. We might, however, thereby overemphasise the element of participation. It is not an equal relationship and some donors will wish to limit their workload and obligations rather than enhance their influence on decision‐making. Rather than emphasising *participation* I believe researchers should ask themselves what type of relationship specific donors have opted for when agreeing to participate’.

Thus even within the biobanking world, a pluralist sociology of donations is needed.

Moving beyond biosamples, other types of contribution, including supplementary information (medical records, cognitive tests, lifestyle questionnaires, etc.), qualitative interviews, clinical trial participation, general time and effort in taking part in research, and even patient involvement in research design, have traditionally and regrettably been omitted from the donation literature. We argue that not only should they be regarded as donations in their own right, but their contribution to more valuable triangulated information that allows for increased scientific endeavour, sometimes over many years, must be acknowledged and further explored. There is an important place for this type of donation, which further demonstrates the need to pluralise current definitions of ‘donation’. The donation is not just the sample, but is so much more – and so much else. The ongoing nature of many research contributions fits with the concept of ‘dynamic consent’ in biobanking (Kaye *et al*. 2015), which provides continuing digital contact with participants and enables them to see and agree to how both samples and data may be re‐used beyond the original purpose if they wish. Kaye *et al*. suggest dynamic consent could extend to other forms of clinical research beyond biobanking; we believe the idea could usefully be applied to narrative social science research too.

In past work, author two has focused on motivations for taking part in clinical trials, and particularly the degree of perceived personal benefit involved, challenging the discourse of altruism, which is so routinely invoked (Locock and Smith 2011a, 2011b). Neat dichotomies between personal benefit and benefiting others – between ‘altruistic’ donation and self‐interest – fail to capture the messiness of real people's motivations. Lucius‐Hoene *et al*. (2013) have shown how the same complexity exists in reasons for narrative research participation. A pluralist sociology of research donations needs to embrace not just hierarchies of different types of donation and varying levels of value attached to them, but also the range of motivations and the shifting boundary between donation and exchange. We suggest that a ‘sociology of research contributions’ might be a more nuanced way to express the mutual and sometimes ongoing relationship between researchers and participants across a range of study types.

## Note

1 Where primary data is used for illustration, we have changed participants’ names.
